# Multimodality Imaging Evaluation of Coronary IgG4-Related Disease: A “Tumor-Like” Cardiac Lesion

**DOI:** 10.3390/diagnostics12112814

**Published:** 2022-11-16

**Authors:** Ludovica R. M. Lanzafame, Maria Ludovica Carerj, Giovanna Rizzo, Fabio Minutoli, Giuseppe M. Bucolo, Natasha Irrera, Giuseppe Muscogiuri, Sandro Sironi, Alfredo Blandino, Tommaso D’Angelo

**Affiliations:** 1 Diagnostic and Interventional Radiology Unit, BIOMORF Department, University Hospital Messina, 98124 Messina, Italy; 2 Centro Cardiologico Monzino IRCCS, 20138 Milan, Italy; 3 Nuclear Medicine Unit, BIOMORF Department, University of Messina, 98124 Messina, Italy; 4 Pharmacology Unit, DIMED Department, University Hospital Messina, 98124 Messina, Italy; 5 Department of Radiology, Istituto Auxologico Italiano IRCCS, San Luca Hospital, 20149 Milan, Italy; 6 Department of Radiology, ASST Papa Giovanni XXIII, 24127 Bergamo, Italy; 7 Department of Radiology and Nuclear Medicine, Erasmus MC, 3015 Rotterdam, The Netherlands

**Keywords:** autoimmune disease, IgG4-associated, IgG4-related disease, cardiac-gated imaging techniques, coronary artery disease, systemic vasculitis

## Abstract

Immunoglobulin G4-related disease (IgG4-RD) is a systemic immune-mediated fibro-inflammatory disorder. Coronary IgG4-RD has been scarcely reported and may present as “tumor-like” lesions. These pseudo-masses may be underdiagnosed mainly due to a vague clinical picture that can vary from complete lack of symptoms to acute coronary syndrome or sudden cardiac death. Early recognition of coronary IgG4-RD is essential to monitor disease activity and prevent life-threatening complications. We report a comprehensive non-invasive imaging evaluation of a patient affected by coronary IgG4-RD, which was diagnosed as an incidental finding during routine pre-laparoscopic cholecystectomy checkup. Non-invasive imaging revealed the presence of a peri-coronary soft-tissue mass that was stable at 12 months follow-up.

## 1. Introduction

Immunoglobulin G4-related disease (IgG4-RD) is an emerging fibro-inflammatory condition with unknown etiology, characterized by lymphoplasmacytic infiltrate rich in IgG4-positive plasma cells, storiform fibrosis, tumor-like lesions and elevated serum IgG4 concentrations in 60–70% of patients [1]. Recently, IgG4-RD has been recognized as a chronic immune-mediated systemic disease, usually affecting middle-aged to elderly patients, that can potentially encompass any organ system. Common presentations include type 1 autoimmune pancreatitis, Mikulicz syndrome, Riedel thyroiditis, sclerosing cholangitis, retroperitoneal fibrosis, interstitial nephritis, prostatitis and lung disease [2].

Periarteritis, with involvement of large-to-medium-sized vessels, may also be a manifestation of IgG4-RD as well as inflammatory aneurysm formation. More rarely, this condition has been reported to affect small-sized vessels such as coronary arteries, leading to potentially life-threatening complications, such as acute coronary syndrome or sudden cardiac death. Histopathologic assessment is considered the method of choice to confirm the diagnosis of IgG4-RD; however, it can be challenging to perform biopsy or to obtain surgical samples when this disease involves vital structures. In these cases, non-invasive imaging evaluation of coronary IgG4-RD may play a crucial role both for the diagnosis and management of this condition [1].

Since IgG4-RD involvement of coronary arteries has been scarcely described, our aim is to report multimodality non-invasive imaging findings of this rare entity and to present a review of the literature.

## 2. Case Presentation

A 63-year-old man came to our observation in May 2021 to perform a chest CT study for suspected pulmonary nodule previously detected on a chest X-ray during a routine pre-laparoscopic cholecystectomy checkup. 

The patient was asymptomatic for angor and dyspnea. Past medical history was significant for multiple comorbidities including obesity, dyslipidemia, arterial hypertension, gouty arthritis, multi-district arthrosis, chronic renal failure, phlebopathy of the lower limbs with dyschromia and trophic ulcerations and multinodular thyroid goiter. The patient reported remote tobacco use, past episodes of chest pain and no history of coronary artery disease.

**Figure 1:**
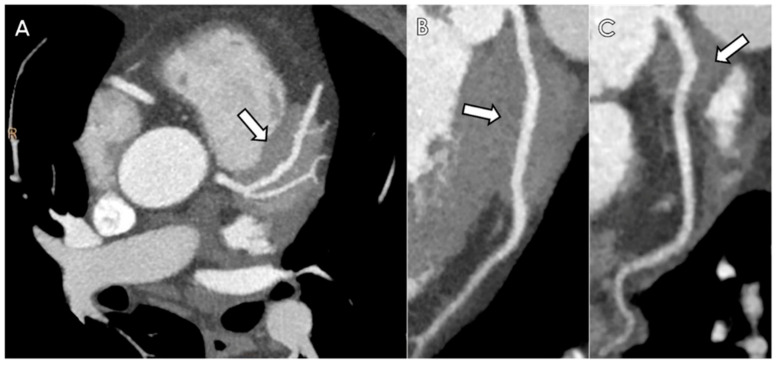
Coronary CTA in a patient with coronary IgG4-RD. Axial maximum intensity projection (**A**) and curved multiplanar reconstructions (**B**,**C**) show the presence of a soft tissue (arrow) surrounding LM, LAD (**A**,**B**) and LCx (**C**) configuring the typical “*pigs-in-a-blanket sign*”.

Chest CT did not reveal lung nodules but a peri-coronary “pseudotumor” as an incidental finding. Dual-energy coronary computed tomography angiography (DE-CCTA) was subsequently performed, and showed a soft-tissue mass within the anterior interventricular sulcus, which encased the distal tract of left main stem (LM), the proximal and the middle segment of the left anterior descending artery (LAD), the first diagonal (D1) and the proximal segment of the left circumflex artery (LCx), without causing any stenosis of the involved coronary segments and configuring the typical “pigs-in-a-blanket” sign (Figure 1). All coronary arteries did not show any intraluminal calcification or stenosis (i.e., Ca score = 0; CAD-RADS 1).

**Figure 2:**
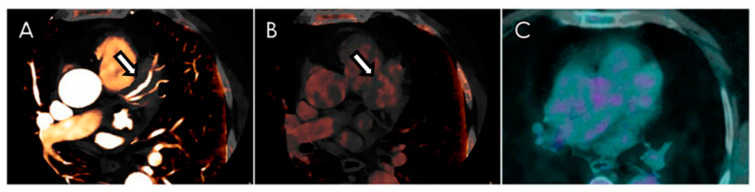
Dual-energy CCTA in a patient with coronary IgG4-RD. Axial iodine map reconstruction does not show any iodine uptake within the pseudo-mass (arrow) during the arterial phase (**A**), while there was a mild increase of iodine concentration during the delayed phase (**B**). Similarly, the low-dose PET-CT investigation acquired 60 min after the administration of 18F-FDG did not show a significant increase of the metabolic tracer (**C**).

The pseudo-mass did not show early enhancement or iodine uptake during arterial phase but had a tenuous late-enhancement at delayed phase (Figure 2A,B). 

Extended laboratory tests revealed mildly elevated IgG (1673 mg/dL—normal range: 700–1600 mg/dL) and IgG4 levels (138 mg/dL—normal range: 5–125 mg/dL) at turbidimetric assays, normal erythrocyte sedimentation rate, normal C-reactive protein levels, normocomplementemia, negative VDRL test and negative rheumatoid factor (RF) and antineutrophil cytoplasmic antibodies (ANCAs). Slightly high serum IgG4 levels raised suspicions of an immune-mediated disease.

**Figure 3:**
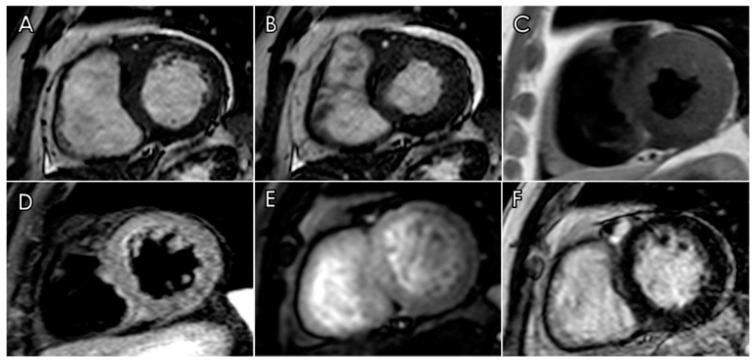
CMR in patient with coronary IgG4-RD. Short axis images performed along the basal slice showing the peri-coronary pseudotumor encasing LAD during end-diastole (**A**) and end-systole (**B**). The lesion appeared isointense to myocardium in balanced steady-state free precession (bSSFP) sequence (**A**,**B**), hypointense in T1-weighted (**C**), and T2-weighted sequences with short-tau inversion recovery (STIR) (**D**). The pseudo-mass did not show any early enhancement during the arterial first-pass (**E**), but late gadolinium enhancement (LGE) was seen in the T1-weighed phase sensitive inversion recovery (PSIR) sequence (**F**).

Cardiac MRI (CMR) was subsequently performed for a non-invasive characterization of the pseudo-mass. The lesion was isointense to myocardium in bSSFP cine-images, heterogeneously hypointense in T2-weighted sequences, and hypointense in T1-weighted sequences. The pseudo-mass did not present early enhancement during arterial first-pass perfusion imaging, but it showed late gadolinium enhancement (LGE), supporting the provisional diagnosis of a fibrotic/granulomatous lesion (Figure 3).

In the suspicion of coronary IgG4-RD, the patient underwent a 18F-fluorodeoxyglucose (FDG) positron-emission tomography (PET) scan, which did not show an increased FDG uptake of the cardiac pseudo-mass (Figure 2C).

In consideration of the complete absence of symptoms, the patency of coronary vessels and the good cardiac function, a biopsy was contraindicated because of the high procedural risk. Moreover, since the non-invasive imaging characteristics of the pseudo-mass were not consistent with active inflammation or malignancy, no surgical or pharmacological treatment was planned.

**Figure 4:**
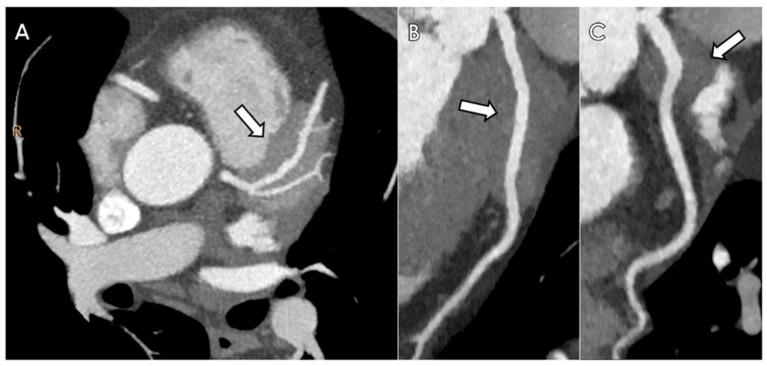
One-year follow-up CCTA in patient with coronary IgG4-RD. Axial maximum intensity projection (**A**) and curved multiplanar reconstructions of LAD (**B**) and LCx (**C**) performed along the same planes of Figure 1. CCTA images show a substantial morpho-volumetric stability of the cardiac pseudo-mass after 12 months (arrow), with the typical “*pigs-in-a-blanket sign*”.

The patient underwent DE-CCTA follow-up studies at 6 months and 12 months, which demonstrated substantial stability of radiological findings and good clinical condition (Figure 4).

## 3. Discussion

IgG4-RD is a systemic immune-mediated inflammatory disease typically associated with high serum IgG4 concentration and IgG4+ plasma cell infiltration that can involve several organs. The most common sites are the pancreas, salivary glands, retroperitoneal organs, and liver [1,3].

Cardiovascular system involvement may manifest with arteritis or periarteritis and inflammatory aneurysms of large-to-middle-size vessels. 

Coronary artery involvement (e.g., stenosis, aneurysm, and diffuse wall thickening) has rarely been reported in the literature, but over the past years its frequency has increased, probably due to the increasing availability of advanced cardiovascular imaging techniques. When this occurs, clinical presentation may vary from complete lack of symptoms to potentially fatal conditions such as acute coronary syndrome or sudden cardiac death [1].

Currently, IgG4-RD diagnosis is defined by the Comprehensive Diagnostic Criteria proposed in 2012 by Umehara et al. and revised in 2020, which refer to specific clinical, radiological, serological and pathological findings [4]. 

Serological findings imply detection of IgG4 serum levels > 135 mg/dL, which were considered critical for the diagnosis of IgG4-RD. Nonetheless, almost 30% of patients shows normal or mildly elevated IgG4 serum and their increase has shown to depend on the number of involved organs, and it is more common in case of pancreatic disease. Increased IgG4 concentration is also not specific for IgG4-RD, since other entities have also been associated with this laboratory finding (e.g., pancreatic adenocarcinoma, lymphoma, ANCA-associated vasculitis, asthma) [5,6].

Biopsy has always been considered the gold standard for the diagnosis of IgG4-RD, since it allows confirmation of the presence of the typical histological features characterized by lymphoplasmacytic infiltrate rich in IgG4-positive plasma cells and storiform fibrosis [7]. According to the AHA/ACC/ESC guidelines, cardiac biopsy is considered the method of choice when diagnosis cannot be determined by non-invasive modalities or when tissue characterization can affect the therapy [8]. However, when lesions are adjacent to vital structures, performing a biopsy might not be an option.

In our case, the lack of clinical symptoms and the benignity of the lesion assessed by comprehensive multimodality imaging suggested a biopsy should not be performed in such a critical anatomical site. As matter of fact, 2019 ACR/EULAR IgG4-RD classification criteria do not require confirmation by biopsy when there is a strong suspicion of IgG4-RD by clinical, serological and radiological findings [9]. 

In our case, coronary CTA allowed for the direct visualization of the soft-tissue mass completely encasing the coronary vessels without causing their luminal narrowing. This finding configures the typical “pigs-in-a-blanket” sign, which is considered to be specific for coronary IgG4-RD [6].

Table 1 summarizes the clinical presentation and the diagnostic workflow of coronary IgG4-RD in all cases reported in the scientific literature, and it emphasizes the increasing role that CCTA has for its diagnosis.

Dual-energy CCTA also provides additional parameters for non-invasive characterization of cardiac lesions [10,11,12,13,14,15]. In our case, it allowed for direct quantification of iodine concentration within the cardiac pseudotumor, both during arterial first-pass and at delayed phase, showing concordant results with CMR. 

CMR findings in coronary IgG4-RD have been scarcely described (Table 1), despite this technique representing the gold standard for non-invasive tissue characterization of cardiac masses [16,17]. CMR has been also used to provide information about disease activity and to guide treatment in vasculitis, such as in Takayasu arteritis [18]. In our case, low signal intensity on both T1- and T2-weighted sequences, together with the absence of early gadolinium enhancement and positive LGE, reflected the fibrotic or granulomatous nature of the lesion. 

Conversely, in case of active perivascular inflammation, increased iodine density during arterial first-pass in DECT or increased signal intensity in T2-weighted sequences and early gadolinium enhancement in CMR would have been expected findings [19]. Moreover, active perivascular inflammation may have led to clinical symptoms such as chest pain or to acute coronary syndrome.

18F-FDG PET/CT represents a remarkable tool to evaluate a systemic inflammatory disease and to highlight IgG4-RD localization for guiding the biopsy, or to monitor treatment response [20]. Its main advantage is to provide metabolic information of the whole body in a single scan. In our case, 18F-FDG PET/CT did not show increased 18F-FDG uptake, suggesting a quiescent phase of coronary IgG4-RD, which was confirmed by follow-up CCTA examinations.

The first-line therapy in patients with symptomatic active IgG4-RD is glucocorticoids, which are extensively used for remission induction and disease relapse, but they can also be used for maintenance therapy. Steroid-sparing agents can also be used for remission maintenance when long-term therapies expose patients to glucocorticoid toxicities. A “watchful waiting” strategy is also suitable in asymptomatic patients and in case of highly fibrotic inactive lesions that are weakly responsive to pharmacologic treatments. In our case, the lack of clinical symptoms, together with laboratory and imaging findings, excluded the presence of active inflammation, and the patient did not require any pharmacological treatment. Moreover, in these patients, the risk/benefit balance may not encourage any therapeutic courses [21].

## 4. Conclusions

Coronary IgG4-RD diagnosis is a rare and potentially fatal condition whose diagnosis is generally challenging. Non-invasive multimodality imaging plays a pivotal role for its diagnosis, since performing a biopsy might not always be an option due to the critical location of these lesions. Finally, non-invasive imaging is crucial to monitor disease activity and guide treatment.

## Figures and Tables

**Table 1:** Literature review of non-invasive/invasive multimodality imaging of coronary pseudo-masses in IgG4-RD.

	Coronary Arteries Involved	Clinical Presentation	TTE	CCTA	CMR	PET-CT	IVUS/OCT	Biopsy
Matsumoto (2008) [22]	RCA	Dyspnea, palpitations	X	X	X			X
Ikutomi (2011) [23]	RCA	Acute coronary syndrome		X				X
Tanigawa (2011) [24]	RCA, LCX	Acute coronary syndrome		X				X
Kusumoto (2012) [25]	RCA, LAD	No symptoms	X	X		Scintigraphy		X
Urabe (2012) [26]	RCA, LCX	Acute coronary syndrome		X		X	X	X
Kan-o (2013) [27]	RCA, LM	Dyspnea		X				
Baruah (2014) [28]	LAD, LCX	Fatigue		X	X			X
Guo (2015) [29]	RCA, LM, LAD, LCX	No symptoms		X		X		
Hourai (2016) [30]	RCA, LAD	Pericardial effusion		X		X		X
Keraliya (2016) [31]	RCA, LM, LAD, LCX	Acute coronary syndrome		X				X
Ito (2016) [32]	LAD, LCX, D1, D2	Chest pain		X			X	
Nishimura (2016) [33]	RCA	No symptoms	X	X				
Kanzaki (2017) [34]	LCX	No symptoms		X		X		X
Komiya (2017) [35]	RCA, LAD	No symptoms	X	X		X		
Sakamoto (2017) [36]	LAD	Palpitations		X				
Rokutanda (2017) [37]	LAD	Chest pain		X				
Matsuda (2018) [38]	LAD, LCX	No symptoms		X		X		
Huang (2018) [20]	LAD, LCX	No symptoms				X		
Okuyama (2019) [39]	RCA, LM, LAD	Acute coronary syndrome		X			X	
Koseki (2019) [40]	RCA, LAD, LCX	No symptoms		X			X	
De la Fuente (2019) [41]	RCA	No symptoms		X				
Ansari-Gilani (2020) [42]	RCA, LAD, LCX	Acute coronary syndrome	X and TEE	X				
Nakamura (2020) [3]	RCA, LAD	No symptoms		X		X	X	
Vasudevan (2021) [43]	LAD.LCX	No symptoms		X		X		
Kubota (2021) [44]	RCA, LAD	No symptoms		X		X		X
Lee (2021) [45]	LAD	No symptoms		X		X		
Yardimci (2021) [46]	LAD, RCA	Chest pain		X				
	RCA	Chest pain, dyspnea		X				X
	RCA, LCX	Chest pain, fatigue		X				
	RCA, LAD, LCX	Chest pain		X				
	RCA, LAD, LCX	Dyspnea, cough, fever		X				
	LAD	No symptoms		X				
Yamaura (2022) [47]	RCA, LAD, LCX	Chest pain	X	X		X		
Maeda (2022) [48]	LAD	Fatigue	X	X		X		X
Liu (2022) [49]	LM, LAD	Palpitations	X	X	X	X		
Ratwatte (2022) [50]	LAD, LCX	Acute coronary syndrome		X				
Karmally (2022) [51]	RCA, LAD	Dyspnea, cough	X	X				
Current Case (2022)	LM, LAD, LCX, D1	No symptoms		X	X	X		

TTE: Transthoracic Echocardiogram; TEE: Transesophageal Echocardiogram; CCTA: Coronary Computed Tomography Angiography; CMR: Cardiovascular Magnetic Resonance; PET-CT: Positron Emission Tomography/Computed Tomography; IVUS: Intravascular Ultrasound; OCT: Optical Coherence Tomography; LAD: left anterior descending artery; LCx: left circumflex artery; LM: left main coronary artery; D1: diagonal 1; D2: diagonal 2; RCA: right coronary artery.

## References

[1] Oyama-Manabe N., Yabusaki S., Manabe O., Kato F., Kanno-Okada H., Kudo K. (2018). IgG4-related Cardiovascular Disease from the Aorta to the Coronary Arteries: Multidetector CT and PET/CT. RadioGraphics.

[2] Sánchez-Oro R., Alonso-Muñoz E.M., Romero L.M. (2019). Review of IgG4-related disease. Gastroenterol. Hepatol..

[3] Nakamura T., Goryo Y., Isojima T., Kawata H. (2021). Immunoglobulin G4-related masses surrounding coronary arteries: A case report. Eur. Heart J.—Case Rep..

[4] Umehara H., Okazaki K., Kawa S., Takahashi H., Goto H., Matsui S., Ishizaka N., Akamizu T., Sato Y., Kawano M. (2021). The 2020 revised comprehensive diagnostic (RCD) criteria for IgG4-RD. Mod. Rheumatol..

[5] Wolfson A.R., Hamilos D.L. (2017). Recent advances in understanding and managing IgG4-related disease. F1000Research.

[6] Wallace Z.S., Deshpande V., Mattoo H., Mahajan V.S., Kulikova M., Pillai S., Stone J.H. (2015). IgG4-Related Disease: Clinical and Laboratory Features in One Hundred Twenty-Five Patients: Clinical And Laboratory Features Of Igg4-Related Disease. Arthritis Rheumatol..

[7] Ramdin N., Orde M., O’Neill S.B., Lai C., Pors J.D., Multan M., Chen L.Y.C., Carruthers M.N. (2021). Hidden IgG4-Related Coronary Disease. Am. J. Clin. Pathol..

[8] Cooper L.T., Baughman K.L., Feldman A.M., Frustaci A., Jessup M., Kuhl U., Levine G.N., Narula J., Starling R.C., Towbin J. (2007). The Role of Endomyocardial Biopsy in the Management of Cardiovascular Disease. J. Am. Coll. Cardiol..

[9] Wallace Z.S., Naden F.R.P., Chari S., Choi H., Della-Torre E., Dicaire J., Hart P.A., Inoue M.D., Khosroshahi A., Kubota K. (2019). The 2019 American College of Rheumatology/European League Against Rheumatism Classification Criteria for IgG4-Related Disease. Ann. Rheum. Dis..

[10] D’Angelo T., Cicero G., Mazziotti S., Ascenti G., Albrecht M.H., Martin S.S., Othman A.E., Vogl T.J., Wichmann J.L. (2019). Dual energy computed tomography virtual monoenergetic imaging: Technique and clinical applications. Br. J. Radiol..

[11] Arendt C.T., Czwikla R., Lenga L., Wichmann J.L., Albrecht M.H., Booz C., Martin S.S., Leithner D., Tischendorf P., Blandino A. (2019). Improved coronary artery contrast enhancement using noise-optimised virtual monoenergetic imaging from dual-source dual-energy computed tomography. Eur. J. Radiol..

[12] Cicero G., Ascenti G., Albrecht M.H., Blandino A., Cavallaro M., D’Angelo T., Carerj M.L., Vogl T.J., Mazziotti S. (2020). Extra-abdominal dual-energy CT applications: A comprehensive overview. La Radiol. medica.

[13] Lenga L., Albrecht M.H., Othman A.E., Martin S.S., Leithner D., D’Angelo T., Arendt C., Scholtz J.-E., De Cecco C.N., Schoepf U.J. (2017). Monoenergetic Dual-energy Computed Tomographic Imaging: Cardiothoracic Applications. J. Thorac. Imaging.

[14] Ascenti G., Sofia C., Mazziotti S., Silipigni S., D’Angelo T., Pergolizzi S., Scribano E. (2016). Dual-energy CT with iodine quantification in distinguishing between bland and neoplastic portal vein thrombosis in patients with hepatocellular carcinoma. Clin. Radiol..

[15] Wang Y., Zhou H., Hu P., Zhao J., Mao Y., Li Z., Zhao X. (2022). Case Report: Dual-Energy Computed Tomography of Cardiac Changes in IgG4-Related Disease. Front. Cardiovasc. Med..

[16] D’Angelo T., Mazziotti S., Inserra M.C., De Luca F., Agati S., Magliolo E., Pathan F., Blandino A., Romeo P. (2019). Cardiac Inflammatory Myofibroblastic Tumor. Circ. Cardiovasc. Imaging.

[17] Koch V., Abt J., Gruenewald L.D., Eichler K., D’Angelo T., Martin S.S., Albrecht M.H., Thalhammer A., Booz C., Yel I. (2022). Systematic evaluation of imaging techniques and baseline characteristics in patients with suspected vasculitis. Eur. J. Radiol. Open.

[18] Ozawa M., Fujinaga Y., Asano J., Nakamura A., Watanabe T., Ito T., Muraki T., Hamano H., Kawa S. (2017). Clinical features of IgG4-related periaortitis/periarteritis based on the analysis of 179 patients with IgG4-related disease: A case–control study. Arthritis Res. Ther..

[19] Patnana M., Sevrukov A.B., Elsayes K.M., Viswanathan C., Lubner M., Menias C.O. (2012). Inflammatory Pseudotumor: The Great Mimicker. Am. J. Roentgenol..

[20] Huang H.L., Fong W., Peh W.M., Niraj K.A., Lam W.W. (2017). The Utility of FDG PET/CT in IgG4-Related Disease with a Focus on Coronary Artery Involvement. Nucl. Med. Mol. Imaging.

[21] Khosroshahi A., Wallace Z.S., Crowe J.L., Akamizu T., Azumi A., Carruthers M.N., Chari S.T., Della-Torre E., Frulloni L., Goto H. (2015). International Consensus Guidance Statement on the Management and Treatment of IgG4-Related Disease: International Consensus Statement On Igg4-Rd Management. Arthritis Rheumatol..

[22] Matsumoto Y., Kasashima S., Kawashima A., Sasaki H., Endo M., Kawakami K., Zen Y., Nakanuma Y. (2008). A case of multiple immunoglobulin G4–related periarteritis: A tumorous lesion of the coronary artery and abdominal aortic aneurysm. Hum. Pathol..

[23] Ikutomi M., Matsumura T., Iwata H., Nishimura G., Ishizaka N., Hirata Y., Ono M., Nagai R. (2011). Giant Tumorous Legions Surrounding the Right Coronary Artery Associated with Immunoglobulin-G4-Related Systemic Disease. Cardiology.

[24] Tanigawa J., Daimon M., Murai M., Katsumata T., Tsuji M., Ishizaka N. (2012). Immunoglobulin G4–related coronary periarteritis in a patient presenting with myocardial ischemia. Hum. Pathol..

[25] Kusumoto S., Kawano H., Takeno M., Kawahara F., Abe K., Hayashi H., Koide Y., Maemura K. (2012). Mass lesions surrounding coronary artery associated with immunoglobulin G4-related disease. J. Cardiol. Cases.

[26] Urabe Y., Fujii T., Kurushima S., Tsujiyama S., Kihara Y. (2012). Pigs-in-a-Blanket Coronary Arteries: A Case of Immunoglobulin G4-Related Coronary Periarteritis Assessed by Computed Tomography Coronary Angiography, Intravascular Ultrasound, and Positron Emission Tomography. Circ. Cardiovasc. Imaging.

[27] Kan-O M., Kado Y., Sadanaga A., Tamiya S., Toyoshima S., Sakamoto M. (2015). Immunoglobulin G4-related multiple cardiovascular lesions successfully treated with a combination of open surgery and corticosteroid therapy. J. Vasc. Surg..

[28] Baruah D., Rubenstein J., Shahir K. (2014). ‘Coronary wrap’: IgG4 related disease of coronary artery presenting as a mass lesion. Int. J. Cardiovasc. Imaging.

[29] Yen A., Guo Y., Ansdell D., Brouha S. (2015). Coronary periarteritis in a patient with multi-organ IgG4-related disease. J. Radiol. Case Rep..

[30] Hourai R., Miyamura M., Tasaki R., Iwata A., Takeda Y., Morita H., Hanaoka N., Tanigawa J., Shibata K., Takeshita A. (2016). A case of IgG4-related lymphadenopathy, pericarditis, coronary artery periarteritis and luminal stenosis. Heart Vessels.

[31] Keraliya A.R., Murphy D.J., Aghayev A., Steigner M.L. (2016). IgG4-Related Disease With Coronary Arteritis. Circ. Cardiovasc. Imaging.

[32] Ito S., Hasuo T., Nimura Y. (2016). iMAP™ imaging of tumorous lesions surrounding the coronary arteries in a patient with an elevated serum level of immunoglobulin G4. Heart Vessels.

[33] Nishimura S., Amano M., Izumi C., Kuroda M., Yoshikawa Y., Takahashi Y., Imamura S., Onishi N., Tamaki Y., Enomoto S. (2016). Multiple Coronary Artery Aneurysms and Thoracic Aortitis Associated with IgG4-related Disease. Intern. Med..

[34] Kanzaki Y., Morita H., Ishizaka N. (2017). Increased 18F-FDG Uptake in IgG4-related Coronary Periarterial Pseudotumor. Intern. Med..

[35] Komiya Y., Soejima M., Tezuka D., Kohsaka H. (2018). Early Detection and Intervention of Coronary Artery Involvement in Immunoglobulin G4-related Disease. Intern. Med..

[36] Sakamoto A., Tanaka T., Hirano K., Koike K., Komuro I. (2017). Immunoglobulin G4-related Coronary Periarteritis and Luminal Stenosis in a Patient with a History of Autoimmune Pancreatitis. Intern. Med..

[37] Rokutanda R., Nishihata Y., Okada M. (2017). IgG4-related Pericoronary Arteritis. J. Rheumatol..

[38] Matsuda J., Takano H., Shimizu W. (2018). IgG4-related periarteritis in the coronary artery and subclinical pericarditis assessed the presence and monitoring of therapy response by PET and CT scan. BMJ Case Rep..

[39] Okuyama T., Tanaka T.D., Nagoshi T., Yoshimura M. (2019). Coronary artery disease concomitant with immunoglobulin G4-related disease: A case report and literature review. Eur. Heart J.—Case Rep..

[40] Koseki K., Yahagi K., Okuno T., Kikushima H., Saito A., Ninomiya K., Tomii D., Nakanishi T., Tanaka T., Sato Y. (2019). Immunoglobulin G4-Related Coronary Periarteritis With Multiple Intracoronary Images. JACC Cardiovasc. Interv..

[41] De La Fuente J., Bird J. (2019). Coronary Arteritis in IgG4-Related Disease. New Engl. J. Med..

[42] Ansari-Gilani K., Gilkeson R.C. (2020). Multimodality imaging of IgG4 related coronary artery aneurysm. Echocardiography.

[43] Vasudevan A.K., Kumar G.A., Rajesh S., Ahamed M.Z. (2021). IgG4-Related Coronary Aneurysm in a Child. Indian J. Pediatr..

[44] Kubota N., Ozaki K., Hoyano M., Okubo T., Kimura S., Yanagawa T., Kashimura T., Inomata T. (2022). Improvement of Mass Lesions around Coronary Arteries and Fractional Flow Reserve after Steroid Therapy in Immunoglobulin-G4-related Coronary Periarteritis. Intern. Med..

[45] Lee N.J., Glockner J.F. (2021). Multisystem IgG4-related disease involving the abdomen and coronary arteries and causing chronic abdominal pain. Acta Radiol. Open.

[46] Yardimci G.K., Duzgun S.A., Bolek E.C., Kilic L., Canpolat U., Hazirolan T., Aytemir K., Karadag O. (2021). Coronary (peri)-arteritis in patients with IgG4-related disease: A case series from the Central Anatolia Region of Turkey. Int. J. Rheum. Dis..

[47] Yamaura H., Ishikawa H., Kasayuki N., Otsuka K. (2022). Morphological and compositional alteration of pericoronary arteritis in a patient with immunoglobulin G4-related disease. Eur. Heart J.—Case Rep..

[48] Maeda R., Naito D., Adachi A., Shiraishi H., Sakamoto T., Matoba S. (2019). IgG4-related Disease Involving the Cardiovascular System: An Intracardiac Mass and a Mass Lesion Surrounding a Coronary Artery. Intern. Med..

[49] Liu X., Zhao Y., Wu N., Zhang W. (2022). Occult myocardial infarction due to an unusual cause: A case report of periarteritis involving the left coronary artery. Eur. Heart J.—Case Rep..

[50] Ratwatte S., Day M., Ridley L.J., Fung C., Naoum C., Yiannikas J. (2022). Cardiac manifestations of IgG4 related disease; a case series. Eur. Heart J.—Case Rep..

[51] Karmally S., Pancholy B., Lau R., Raparia K., Pursnani S. (2022). IgG4-related disease: Coronary arteritis masquerading as coronary “masses”. J. Cardiovasc. Comput. Tomogr..

